# Prognostic Role of the C-PLAN Index in Metastatic Melanoma: A Two-Center Retrospective Study

**DOI:** 10.3390/jcm15218261

**Published:** 2026-10-23

**Authors:** İrfan Buğday, Safiye Selcen Vicdanlı, Mevlüde İnanç, Metin Özkan, Oktay Bozkurt

**Affiliations:** 1 Medical Oncology Department, Kayseri City Hospital, 38080 Kayseri, Türkiye; 2 Medical Oncology Department, Erciyes University Medical School, 38030 Kayseri, Türkiye; selcengokturk@gmail.com (S.S.V.);

**Keywords:** C-PLAN index, melanoma, metastatic melanoma, prognosis, progression-free survival, overall survival, systemic inflammation

## Abstract

**Background:** The C-PLAN index is a composite prognostic tool incorporating clinical, inflammatory, nutritional, and biochemical parameters. Its prognostic role in metastatic melanoma remains insufficiently investigated. This study aimed to evaluate the association between the C-PLAN index and survival outcomes in patients with metastatic melanoma. **Methods:** This retrospective two-center study included 130 patients with metastatic melanoma treated at Erciyes University and Erzurum City Hospital between January 2012 and September 2023. The C-PLAN index was calculated using five parameters: Eastern Cooperative Oncology Group performance status, C-reactive protein, lactate dehydrogenase, serum albumin, and derived neutrophil-to-lymphocyte ratio (dNLR). Patients were classified into C-PLAN score groups of 0–1 and ≥2. Progression-free survival (PFS) and overall survival (OS) were evaluated using Kaplan–Meier analysis and Cox proportional hazards regression. **Results:** Among the 130 patients, 48 (36.9%) had a C-PLAN score of 0–1 and 82 (63.1%) had a score of ≥2. During follow-up, 76 patients experienced a PFS event, and 94 died. Median PFS was longer in the C-PLAN 0–1 group than in the ≥2 group (30.27 vs. 9.17 months; log-rank *p* < 0.001). Median OS was also longer in the C-PLAN 0–1 group (82.93 vs. 29.70 months; log-rank *p* < 0.001). In the multivariable Cox regression analysis for PFS, a C-PLAN score of ≥2 was associated with a higher hazard of progression or death (HR, 3.193; 95% CI, 1.774–5.746; *p* < 0.001). A higher number of metastatic sites, BMI category, mitotic activity, lymphatic invasion, and BRAF status were also independently associated with PFS. In the multivariable Cox regression analysis for OS, a C-PLAN score of ≥2 (HR, 3.227; 95% CI, 1.910–5.455; *p* < 0.001) and a higher number of metastatic sites (HR, 2.453; 95% CI, 1.538–3.912; *p* < 0.001) were independently associated with a higher hazard of death. **Conclusions:** In this retrospective two-center cohort, a C-PLAN score of ≥2 was associated with shorter PFS and OS. These findings should be interpreted as exploratory and support further investigation of C-PLAN as a prognostic index in melanoma. Validation in larger, contemporary, and independent cohorts is required before its clinical utility can be established.

## 1. Introduction

Melanoma is a malignant tumor arising from melanocytes and is one of the most aggressive forms of skin cancer. Ultraviolet (UV) radiation, particularly excessive and intermittent sun exposure and sunburn, is an important environmental risk factor for melanoma development. Recent systematic evidence has further supported the association between UV exposure and melanoma risk, particularly in individuals with fairer skin types [1]. Although many melanomas are diagnosed at an early stage and can be successfully treated with surgical excision, some patients present with metastatic disease or subsequently develop metastases during follow-up. Once melanoma becomes metastatic, prognosis worsens substantially, and survival outcomes remain heterogeneous among patients with advanced disease. Therefore, the identification of reliable and readily available prognostic factors remains clinically important.

The development and progression of melanoma are driven by multiple molecular alterations involving signaling pathways that regulate cell proliferation, survival, and immune interactions. Genomic analyses have classified cutaneous melanoma into molecular subtypes characterized predominantly by alterations in BRAF, RAS, and NF1, as well as tumors lacking these major driver alterations [2]. Among these, BRAF mutations, particularly BRAF V600 mutations, are of major clinical importance because constitutive activation of the mitogen-activated protein kinase (MAPK) pathway promotes tumor cell proliferation and provides a therapeutic target. The identification of these molecular alterations has led to the development of effective targeted therapies for patients with BRAF-mutant melanoma. In parallel, immune checkpoint inhibitors targeting programmed cell death protein 1 (PD-1) and cytotoxic T-lymphocyte-associated antigen 4 (CTLA-4) have substantially improved outcomes in advanced melanoma and have become central components of systemic treatment [3]. Despite these therapeutic advances, considerable heterogeneity in treatment response and survival persists, emphasizing the continued need for clinically accessible prognostic markers.

Several clinical and pathological characteristics have been associated with prognosis in metastatic melanoma. Performance status, serum lactate dehydrogenase (LDH), and the extent and distribution of metastatic disease are among the factors that have consistently been associated with survival outcomes [4]. In particular, poorer performance status, elevated LDH levels, and a greater number of metastatic sites have been associated with worse survival. However, these parameters primarily reflect tumor burden and the general clinical condition of the patient and may not fully capture the systemic inflammatory and nutritional status associated with cancer progression. A prognostic approach that integrates clinical status with systemic biological characteristics may therefore provide additional prognostic information.

Systemic inflammation has increasingly been recognized as an important component of cancer progression and prognosis. Several routinely available hematological and biochemical parameters, including the neutrophil-to-lymphocyte ratio (NLR), platelet-to-lymphocyte ratio, lymphocyte-to-monocyte ratio, systemic immune–inflammation index, C-reactive protein (CRP), albumin, and LDH, have been investigated as potential prognostic biomarkers in melanoma. Among these markers, NLR has been particularly well studied. A recent systematic review and meta-analysis including 37 studies demonstrated that elevated NLR was associated with both shorter overall survival (OS) and progression-free survival (PFS) in patients with melanoma [5]. Similarly, a recent meta-analysis of 19 studies involving 4634 patients reported that elevated CRP was associated with poorer OS and PFS [6]. In addition to these routinely available markers, circulating microRNAs have also been investigated as potential prognostic biomarkers in melanoma, although their clinical application requires further validation [7,8]. These findings support the potential prognostic relevance of systemic inflammatory, nutritional, and molecular biomarkers in melanoma.

Several composite prognostic indices have consequently been developed to integrate multiple inflammatory, nutritional, and clinical characteristics. The modified Glasgow Prognostic Score (mGPS), which incorporates CRP and serum albumin, has demonstrated prognostic value across a wide range of malignancies [9]. The Lung Immune Prognostic Index (LIPI), which combines derived NLR (dNLR) and LDH, was developed as an inflammation-based prognostic tool and has subsequently been investigated in patients receiving immune checkpoint inhibitors [10]. Although such indices provide a more comprehensive assessment than individual biomarkers, different scores incorporate different aspects of systemic inflammation and clinical status. In particular, performance status, which reflects the functional condition of the patient and is an established prognostic factor in metastatic melanoma, is not included in mGPS or LIPI.

The C-PLAN index is a composite prognostic index incorporating CRP, performance status (PS), LDH, albumin, and dNLR. In its original formulation, the inflammatory component was dNLR, and the index was initially investigated in patients with advanced lung cancer receiving immune checkpoint inhibitor-based treatment [11]. Subsequent studies have also reported prognostic associations between C-PLAN and survival in other advanced lung cancer populations [12]. By combining clinical performance status with inflammatory, nutritional, and biochemical parameters, C-PLAN provides a simple approach based on routinely available clinical and laboratory data. However, its prognostic role in melanoma has not been established.

Therefore, this two-center retrospective study aimed to evaluate the association between the C-PLAN index and PFS and OS in patients with metastatic melanoma. The cohort included patients with cutaneous melanoma as well as uveal and rectal primary tumors; this histological heterogeneity should be considered when interpreting the findings. We hypothesized that a higher C-PLAN score would be associated with poorer clinical outcomes. To our knowledge, this is the first study to evaluate the prognostic role of the C-PLAN index in patients with melanoma.

## 2. Materials and Methods

### 2.1. Study Design and Patient Population

This retrospective two-center study included patients with metastatic malignant melanoma treated at the Department of Medical Oncology, Erciyes University, and Erzurum City Hospital between 1 January 2012 and 30 September 2023. Patients with metastatic disease at diagnosis and those who developed metastatic disease during follow-up were eligible for inclusion. Laboratory parameters used to calculate the C-PLAN index were assessed when there was no clinical evidence of infection.

Clinical, pathological, laboratory, treatment, and survival data were retrospectively obtained from institutional electronic medical records and hospital databases. The collected variables included age, sex, primary tumor characteristics, pathological features, metastatic sites, treatment characteristics, treatment response, adverse events, and survival outcomes. The data cutoff date was 30 September 2023. All 130 patients were included in the survival analyses. Data availability varied for individual treatment-related adverse-event variables.

### 2.2. Treatment and Response Assessment

First-line systemic treatment for metastatic disease included temozolomide, dabrafenib plus trametinib, or ipilimumab plus nivolumab, according to treatment practices and available therapeutic options during the study period. Tumor response was assessed according to the Response Evaluation Criteria in Solid Tumors (RECIST), version 1.1. The best overall response obtained during first-line treatment for metastatic disease was recorded.

### 2.3. Definition and Calculation of the C-PLAN Index

The C-PLAN index was calculated using five routinely available clinical and laboratory parameters: Eastern Cooperative Oncology Group performance status (ECOG PS), C-reactive protein (CRP), lactate dehydrogenase (LDH), serum albumin, and the derived neutrophil-to-lymphocyte ratio (dNLR). One point was assigned for each of the following criteria: ECOG PS ≥ 2, CRP above the laboratory upper limit of normal (ULN), LDH above the ULN, serum albumin < 3.5 g/dL, and dNLR ≥ 3. The total C-PLAN score ranged from 0 to 5. The dNLR was calculated as the neutrophil count divided by the difference between the total white blood cell count and the neutrophil count.

For survival analyses, patients were categorized into two groups according to their C-PLAN score: 0–1 and ≥2.

### 2.4. Survival Outcomes

Progression-free survival (PFS) was defined as the time from initiation of first-line systemic treatment for metastatic disease to radiologically or clinically documented disease progression or death from any cause, whichever occurred first. Patients without documented progression or death at the last follow-up were censored on that date.

Overall survival (OS) was defined as the time from initiation of first-line systemic treatment for metastatic disease to death from any cause. Patients who were alive at the last follow-up were censored on the date of their last documented follow-up.

### 2.5. Statistical Analysis

Statistical analyses were performed using IBM SPSS Statistics, version 25.0 (IBM Corp., Armonk, NY, USA). Continuous variables were summarized using mean ± standard deviation or median and range, as appropriate, while categorical variables were reported as frequencies and percentages.

Kaplan–Meier methods were used to estimate PFS and OS, and survival distributions between C-PLAN groups were compared using the log-rank test. Univariable and multivariable Cox proportional hazards regression analyses were performed to evaluate potential prognostic factors for PFS and OS. The primary multivariable models included the C-PLAN group, number of metastatic sites, mitotic activity, BRAF V600 mutation status (categorized as positive or negative), body mass index (BMI) category, and lymphatic invasion.

A sensitivity analysis was performed in patients with cutaneous melanoma, excluding those with uveal and rectal melanoma, to assess whether the association between the C-PLAN group and PFS remained consistent in the cutaneous melanoma population.

Additional multivariable analyses were performed to account for study center, liver metastasis, brain metastasis, treatment year, and first-line treatment type. Hazard ratios (HRs) with 95% confidence intervals (CIs) were reported.

The proportional hazards assumption was assessed using Schoenfeld residuals in R, version 4.6.1. All statistical tests were two-sided, and a *p*-value < 0.05 was considered statistically significant.

To explore the incremental prognostic contribution of the C-PLAN index, the discriminative performance of a reference Cox model containing metastatic site count, mitotic activity, BRAF status, BMI category, and lymphatic invasion was compared with that of a model additionally including the C-PLAN group. Model discrimination was assessed using Harrell’s concordance index (C-index). Internal validation was performed using 1000 bootstrap resamples, and optimism-corrected C-index estimates were obtained. These analyses were considered exploratory.

In supplementary analyses, age group and sex were additionally included in the multivariable Cox regression models to assess the robustness of the associations between C-PLAN and PFS and OS after further adjustment. These analyses were considered supplementary and did not replace the primary multivariable models.

### 2.6. Ethical Considerations

The study protocol was approved by the Ethics Committee of Erciyes University Faculty of Medicine on 4 October 2023 (approval number: 2023/654). The study was conducted in accordance with the principles of the Declaration of Helsinki.

Given the retrospective design and anonymized data collection process, the requirement for informed consent was waived by the ethics committee.

## 3. Results

### 3.1. Patient Characteristics

A total of 130 patients with metastatic malignant melanoma were included in the study. The median age was 61 years (range, 24–88 years), and 74 patients (56.9%) were male. The distribution of disease stages at initial diagnosis (stages I, II, III, and IV) was 4 (3.1%), 46 (35.4%), 46 (35.4%), and 34 (26.2%) patients, respectively. BRAF V600 mutations were detected in 43 patients (33.1%), whereas 87 patients (66.9%) had wild-type BRAF V600. Lung, liver, brain, and bone metastases were present in 71, 35, 35, and 25 patients, respectively. Primary tumor locations included the head and neck (n = 43), trunk (n = 22), upper extremities (n = 15), lower extremities (n = 36), uvea (n = 7), and rectum (n = 7).

The number of metastatic sites was ≤2 in 95 patients and >2 in 35 patients. Mitotic activity was <5 in 65 patients and ≥5 in 65 patients. Lymphatic invasion was absent in 57 patients and present in 73 patients. First-line systemic treatments consisted of temozolomide in 64 patients, dabrafenib plus trametinib in 32 patients, and ipilimumab plus nivolumab in 34 patients. The baseline demographic, clinical, and pathological characteristics of the study population, including the five components of the C-PLAN index, are summarized in Table 1.

### 3.2. Treatment Response and Treatment-Related Adverse Events

Regarding the best overall response to first-line treatment for metastatic disease, complete response was observed in 8 patients (6.2%), partial response in 44 (33.8%), stable disease in 53 (40.8%), and progressive disease in 25 patients (19.2%). Recorded treatment-related adverse events included neutropenia in 7 of 129 patients (5.4%), thrombocytopenia in 6 (4.7%), mucositis in 2 of 128 (1.6%), diarrhea in 4 of 129 (3.1%), rash in 2 (1.6%), and pneumonitis in 1 patient (0.8%). The denominators reflect the available data for each adverse-event variable.

### 3.3. C-PLAN Distribution and Survival Outcomes

Among the 130 patients, 48 (36.9%) had a C-PLAN score of 0–1, whereas 82 (63.1%) had a score of ≥2.

**Figure 1:**
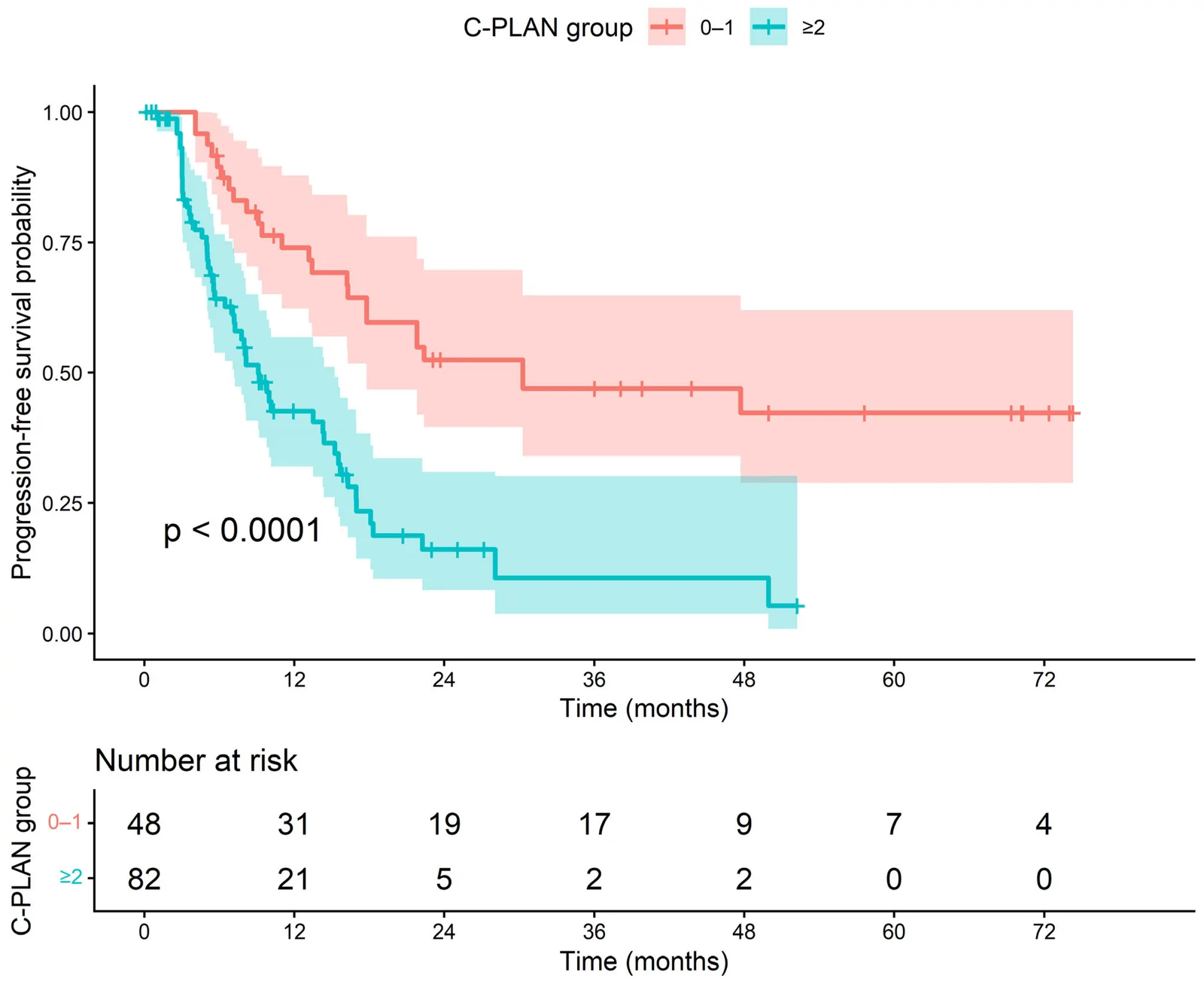
Progression-free survival according to C-PLAN groups. Kaplan–Meier curves showing progression-free survival in patients with C-PLAN scores of 0–1 and ≥2. The difference between the groups was statistically significant (log-rank test, χ^2^ = 18.544, *p* < 0.001).

The median progression-free survival (PFS) was 30.267 months (95% CI, 15.369–60.390) in patients with a C-PLAN score of 0–1 and 9.167 months (95% CI, 7.011–11.322) in those with a score of ≥2. The difference between the groups was statistically significant (log-rank χ^2^ = 18.544, *p* < 0.001; Figure 1).

**Figure 2:**
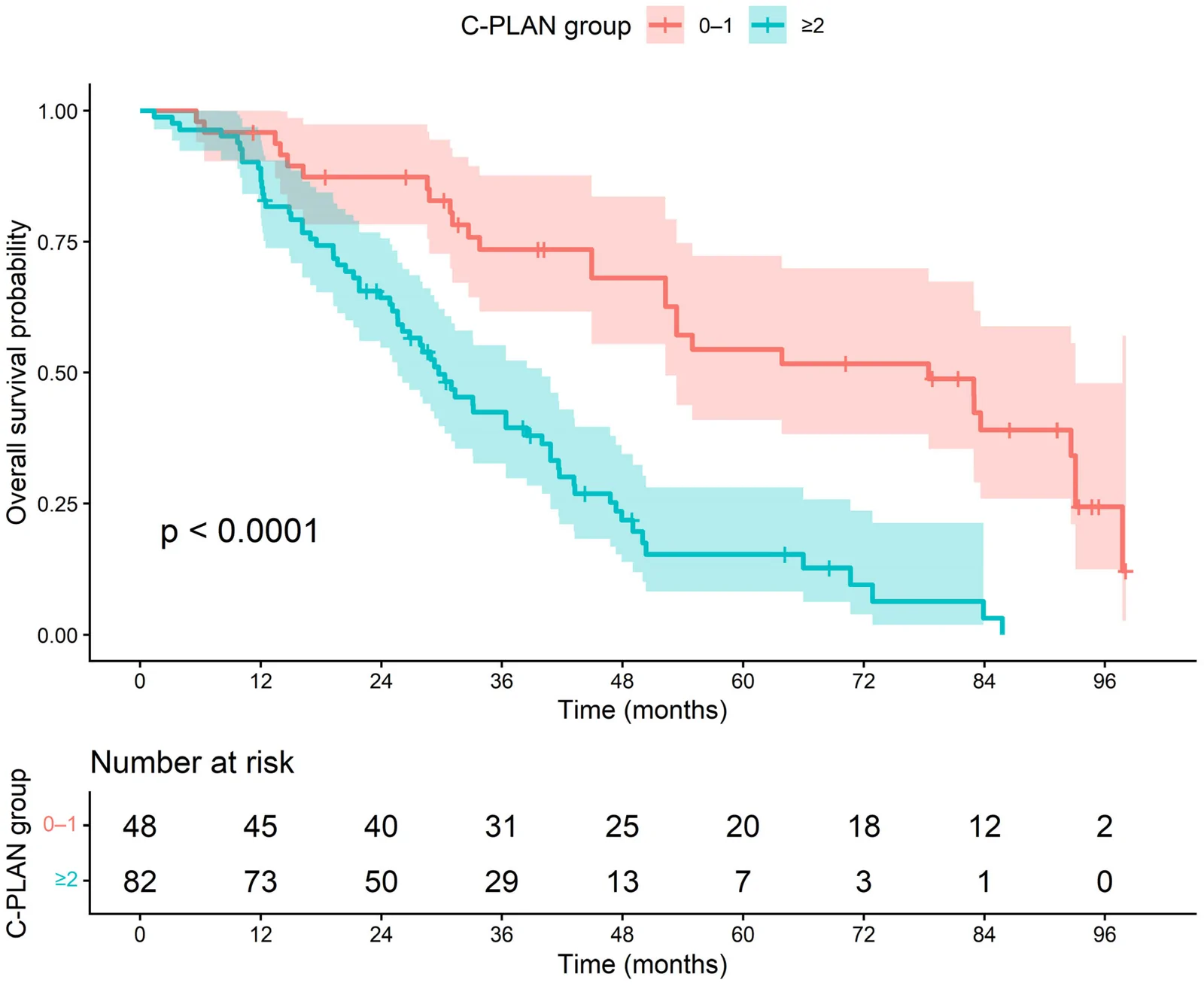
Overall survival according to C-PLAN groups. Kaplan–Meier curves showing overall survival in patients with C-PLAN scores of 0–1 and ≥2. The difference between the groups was statistically significant (log-rank test, χ^2^ = 28.895, *p* < 0.001).

The median overall survival (OS) was 82.933 months (95% CI, 44.942–120.925) in the C-PLAN 0–1 group and 29.700 months (95% CI, 24.602–34.798) in the C-PLAN ≥2 group. This difference was also statistically significant (log-rank χ^2^ = 28.895, *p* < 0.001; Figure 2). The median OS for the overall study population was 40.833 months (95% CI, 32.871–48.796). The median follow-up duration, estimated using the reverse Kaplan–Meier method, was 98.1 months (95% CI, 74.2–122.0).

### 3.4. Univariable Cox Regression Analysis

In the univariable Cox regression analysis for PFS, a C-PLAN score of ≥2 was associated with a significantly higher hazard of progression or death than a score of 0–1 (HR, 2.897; 95% CI, 1.749–4.797; *p* < 0.001). A metastatic site count of >2 (HR, 3.818; 95% CI, 2.270–6.423; *p* < 0.001), lymphatic invasion (HR, 3.676; 95% CI, 2.274–5.942; *p* < 0.001), mitotic activity ≥ 5 (HR, 2.206; 95% CI, 1.393–3.494; *p* = 0.001), BRAF status (HR, 1.807; 95% CI, 1.102–2.962; *p* = 0.019), and BMI category (HR, 2.290; 95% CI, 1.344–3.902; *p* = 0.002) were also significantly associated with PFS. Liver metastasis (HR, 3.138; 95% CI, 1.910–5.157; *p* < 0.001) and brain metastasis (HR, 2.404; 95% CI, 1.470–3.930; *p* < 0.001) were associated with a higher hazard of progression or death. First-line treatment type was also associated with PFS (overall *p* = 0.012), whereas treatment year (HR, 1.071; 95% CI, 0.959–1.198; *p* = 0.224) and study center (HR, 1.000; 95% CI, 0.559–1.791; *p* = 0.999) were not significantly associated with PFS.

In the univariable Cox regression analysis for OS, a C-PLAN score of ≥2 was associated with a higher hazard of death (HR, 3.713; 95% CI, 2.242–6.148; *p* < 0.001). A metastatic site count of >2 (HR, 3.939; 95% CI, 2.445–6.346; *p* < 0.001), lymphatic invasion (HR, 1.983; 95% CI, 1.303–3.018; *p* = 0.001), mitotic activity ≥ 5 (HR, 1.673; 95% CI, 1.108–2.528; *p* = 0.014), liver metastasis (HR, 1.595; 95% CI, 1.007–2.525; *p* = 0.047), and brain metastasis (HR, 2.140; 95% CI, 1.377–3.325; *p* = 0.001) were also significantly associated with OS. BRAF status (HR, 1.224; 95% CI, 0.788–1.902; *p* = 0.368), BMI category (HR, 1.357; 95% CI, 0.875–2.104; *p* = 0.173), treatment year (HR, 1.011; 95% CI, 0.921–1.109; *p* = 0.821), study center (HR, 1.315; 95% CI, 0.801–2.158; *p* = 0.279), and first-line treatment type (overall *p* = 0.786) were not significantly associated with OS.

### 3.5. Distribution of Individual C-PLAN Scores and Exploratory Survival Analyses

The distribution of individual C-PLAN scores among the 130 patients was as follows: score 0, 20 patients (15.4%); score 1, 28 patients (21.5%); score 2, 26 patients (20.0%); score 3, 26 patients (20.0%); score 4, 21 patients (16.2%); and score 5, 9 patients (6.9%). In exploratory Kaplan–Meier analyses, survival distributions differed significantly across the six C-PLAN score categories for OS (log-rank χ^2^ = 44.641, df = 5, *p* < 0.001) and PFS (log-rank χ^2^ = 40.442, df = 5, *p* < 0.001). However, survival outcomes did not follow a strictly monotonic pattern across increasing scores. The predefined comparison of C-PLAN scores of 0–1 versus ≥2 remained the primary analysis.

### 3.6. Primary Multivariable Cox Regression Analyses

In the primary multivariable Cox regression model for PFS, the overall model was statistically significant (χ^2^ = 76.152, df = 6, *p* < 0.001). A C-PLAN score of ≥2 remained independently associated with a higher hazard of progression or death (HR, 3.193; 95% CI, 1.774–5.746; *p* < 0.001). A metastatic site count of >2 (HR, 3.482; 95% CI, 1.989–6.093; *p* < 0.001), BMI category (HR, 2.159; 95% CI, 1.243–3.753; *p* = 0.006), mitotic activity ≥ 5 (HR, 2.238; 95% CI, 1.305–3.839; *p* = 0.003), lymphatic invasion (HR, 3.165; 95% CI, 1.856–5.398; *p* < 0.001), and BRAF status (HR, 2.060; 95% CI, 1.225–3.465; *p* = 0.006) were also independently associated with PFS.

In the primary multivariable Cox regression model for OS, the overall model was statistically significant (χ^2^ = 48.924, df = 6, *p* < 0.001). A C-PLAN score of ≥2 (HR, 3.227; 95% CI, 1.910–5.455; *p* < 0.001) and a metastatic site count of >2 (HR, 2.453; 95% CI, 1.538–3.912; *p* < 0.001) remained independently associated with OS. BMI category (HR, 0.995; 95% CI, 0.626–1.581; *p* = 0.983), mitotic activity (HR, 1.257; 95% CI, 0.785–2.011; *p* = 0.341), lymphatic invasion (HR, 1.235; 95% CI, 0.813–1.874; *p* = 0.322), and BRAF status (HR, 1.058; 95% CI, 0.660–1.694; *p* = 0.816) were not independently associated with OS. The results of the primary multivariable analyses are presented in Table 2.

### 3.7. Additional Multivariable Analyses

Additional multivariable Cox regression models were fitted to account for study center, liver metastasis, brain metastasis, treatment year, and first-line treatment type, in addition to the six variables included in the primary models.

The expanded PFS model was statistically significant (χ^2^ = 107.342, df = 12, *p* < 0.001; −2 log likelihood = 524.615). A C-PLAN score of ≥2 remained independently associated with a higher hazard of progression or death (HR, 2.889; 95% CI, 1.614–5.171; *p* < 0.001). A metastatic site count of >2 (HR, 2.155; 95% CI, 1.062–4.375; *p* = 0.033), lymphatic invasion (HR, 4.979; 95% CI, 2.647–9.362; *p* < 0.001), mitotic activity ≥ 5 (HR, 2.159; 95% CI, 1.248–3.734; *p* = 0.006), BMI category (HR, 2.043; 95% CI, 1.140–3.661; *p* = 0.016), and brain metastasis (HR, 2.601; 95% CI, 1.370–4.939; *p* = 0.003) remained significantly associated with PFS. Study center, BRAF status, liver metastasis, treatment year, and first-line treatment type were not statistically significant in this expanded model.

The expanded OS model was also statistically significant (χ^2^ = 64.131, df = 12, *p* < 0.001; −2 log likelihood = 702.053). A C-PLAN score of ≥2 (HR, 3.013; 95% CI, 1.751–5.184; *p* < 0.001), a metastatic site count of >2 (HR, 3.535; 95% CI, 1.907–6.553; *p* < 0.001), and lymphatic invasion (HR, 1.729; 95% CI, 1.042–2.868; *p* = 0.034) were independently associated with OS. Study center, BRAF status, mitotic activity, BMI category, liver metastasis, brain metastasis, treatment year, and first-line treatment type were not statistically significant in this expanded model.

In supplementary multivariable Cox regression models additionally adjusted for age group and sex, C-PLAN remained independently associated with both PFS (HR, 3.588; 95% CI, 1.983–6.492; *p* < 0.001) and OS (HR, 2.683; 95% CI, 1.567–4.595; *p* < 0.001). In the PFS model, metastatic-site count, BRAF status, lymphatic invasion, mitotic activity, BMI group, and sex were also significantly associated with PFS, whereas age group was not (*p* = 0.404). In the OS model, metastatic-site count and age group were significantly associated with OS, whereas BRAF status, lymphatic invasion, mitotic activity, BMI group, and sex were not statistically significant.

In the exploratory assessment of model discrimination, the C-index was 0.787 for the reference model and 0.813 for the model additionally including C-PLAN (difference, 0.026; bootstrap 95% CI, 0.002–0.057). After bootstrap-based optimism correction using 1000 resamples, the corresponding C-index estimates were 0.776 and 0.802, respectively, with an optimism-corrected difference of 0.025. The likelihood-ratio test indicated improved model fit after adding C-PLAN (χ^2^ = 14.943, df = 1, *p* < 0.001). These exploratory findings suggest a potential incremental contribution of C-PLAN to model discrimination.

### 3.8. Sensitivity Analysis in Cutaneous Melanoma

In a sensitivity analysis restricted to patients with cutaneous melanoma, excluding those with uveal and rectal melanoma, the association between the C-PLAN group and PFS remained statistically significant. The median PFS was 30.27 months (95% CI, 0.00–67.80) in the C-PLAN 0–1 group and 9.17 months (95% CI, 3.53–14.80) in the C-PLAN ≥ 2 group (log-rank χ^2^ = 16.307, *p* < 0.001).

### 3.9. Assessment of the Proportional Hazards Assumption

The proportional hazards assumption was assessed using Schoenfeld residuals in R. For the primary PFS model, no statistically significant departures from the proportional hazards assumption were observed for the individual covariates or the global test (global *p* = 0.060). For the primary OS model, the global test was not statistically significant (*p* = 0.057); however, mitotic activity (*p* = 0.021) and BRAF status (*p* = 0.032) showed potential departures from proportional hazards. These findings were considered when interpreting the primary OS model results.

## 4. Discussion

In this two-center retrospective study of patients with metastatic malignant melanoma, the C-PLAN index was significantly associated with both progression-free survival (PFS) and overall survival (OS). Patients with a C-PLAN score of 0–1 had substantially longer PFS and OS than those with a score of ≥2. Importantly, the prognostic association of C-PLAN remained significant after adjustment for other clinical and pathological variables in the primary multivariable Cox regression analyses and in additional models incorporating study center, liver metastasis, brain metastasis, treatment year, and first-line treatment type. In addition to C-PLAN, the number of metastatic sites was independently associated with both PFS and OS in the primary models, whereas mitotic activity, BRAF status, BMI group, and lymphatic invasion were associated with PFS but not independently associated with OS in these models.

The C-PLAN index was originally developed as a composite prognostic tool incorporating C-reactive protein (CRP), performance status (PS), lactate dehydrogenase (LDH), albumin, and derived neutrophil-to-lymphocyte ratio (dNLR). In the original study of advanced lung cancer, higher C-PLAN scores were associated with shorter PFS and OS and remained independently associated with survival after multivariable adjustment [9]. A subsequent study in metastatic renal cell carcinoma also reported a significant prognostic association between C-PLAN and survival outcomes [13]. Our findings extend these observations to metastatic melanoma, in which C-PLAN was independently associated with both PFS and OS. These results suggest that the prognostic relevance of C-PLAN may extend beyond the malignancies in which it has previously been investigated, although external validation is required before its clinical utility in melanoma can be established.

The biological rationale for combining these parameters is supported by the melanoma literature. LDH has long been recognized as an important prognostic marker in metastatic melanoma, while performance status and the extent of metastatic disease have also been consistently associated with survival. In a pooled analysis of 1362 patients enrolled in Eastern Cooperative Oncology Group trials, performance status, LDH, and the number of metastatic sites were among the factors associated with survival [4]. More recent evidence has further supported the prognostic relevance of systemic inflammatory biomarkers in melanoma. A 2025 systematic review and meta-analysis including 37 studies found that elevated neutrophil-to-lymphocyte ratio (NLR) was associated with inferior OS and PFS in patients with melanoma [5]. Similarly, a meta-analysis of 19 studies including 4634 patients found that elevated CRP was associated with shorter OS and PFS in melanoma [6]. Together, these findings provide a rationale for integrating inflammatory biomarkers such as CRP and dNLR with LDH, albumin, and performance status into a composite prognostic index.

In our cohort, the number of metastatic sites was independently associated with both PFS and OS in the primary multivariable analyses. This finding is consistent with previous evidence demonstrating the prognostic importance of metastatic disease burden in advanced melanoma. In the pooled analysis of Eastern Cooperative Oncology Group trials, the extent of metastatic disease was associated with survival outcomes [4]. The persistence of metastatic burden as an independent factor in our multivariable models suggests that C-PLAN and anatomical disease extent may provide complementary prognostic information. Importantly, metastatic burden is not incorporated into the C-PLAN score itself, which may explain why both variables retained independent associations with survival.

Mitotic activity was independently associated with PFS in our primary multivariable analysis but not with OS. Mitotic rate is an established marker of melanoma biology, and the American Joint Committee on Cancer staging system recognizes its prognostic relevance in melanoma [14]. The association between mitotic activity and PFS in our study may reflect the contribution of tumor proliferative activity to disease progression. The lack of a statistically significant independent association with OS, however, may indicate that overall survival is influenced by additional factors after progression, including subsequent systemic treatments, treatment sequencing, and the clinical course of metastatic disease. This finding should also be interpreted in the context of the potential departure from the proportional hazards assumption identified for mitotic activity in the OS model.

BRAF status, BMI group, and lymphatic invasion were independently associated with PFS in the primary multivariable analysis but were not independently associated with OS. These differences illustrate that factors associated with disease progression do not necessarily retain the same prognostic association with overall mortality. BRAF status has an established role in treatment selection in metastatic melanoma, and clinical guidelines recommend BRAF V600 testing because BRAF/MEK-targeted therapy is an important treatment option for patients with BRAF V600-mutant disease [3]. In a retrospective cohort spanning different treatment approaches and treatment eras, the relationship between BRAF status and OS may consequently be influenced by subsequent treatment exposure and treatment sequencing. Therefore, the absence of a statistically significant independent OS association for BRAF in our analysis should not be interpreted as evidence that BRAF status lacks clinical importance. In addition, the potential departure from the proportional hazards assumption observed for BRAF status in the OS model warrants cautious interpretation of its estimated hazard ratio.

The additional multivariable analyses provided further information about the robustness of the observed associations. After adjustment for study center, liver metastasis, brain metastasis, treatment year, and first-line treatment type, C-PLAN remained significantly associated with both PFS and OS. The number of metastatic sites also remained significantly associated with both endpoints, while lymphatic invasion was additionally associated with OS in the expanded model. In the expanded PFS model, brain metastasis was significantly associated with PFS, whereas liver metastasis, study center, treatment year, and first-line treatment type were not statistically significant. These findings support the persistence of the association between C-PLAN and survival after accounting for additional clinical and treatment-related factors. Nevertheless, these analyses remain observational and cannot exclude residual confounding or establish that C-PLAN provides incremental predictive performance beyond established prognostic factors.

### Sensitivity Analysis in Cutaneous Melanoma

In the sensitivity analysis restricted to patients with cutaneous melanoma, excluding those with uveal and rectal melanoma, the association between the C-PLAN group and progression-free survival remained statistically significant. This finding suggests that the observed prognostic association was not solely attributable to the inclusion of these less common melanoma primary sites. However, this analysis was limited to progression-free survival, and the findings require confirmation in independent cohorts.

In an exploratory assessment of incremental model discrimination, adding C-PLAN to the reference model increased the C-index from 0.787 to 0.813. After bootstrap-based optimism correction, the corresponding estimates were 0.776 and 0.802, respectively. Although these findings suggest a potential improvement in model discrimination, the magnitude and clinical relevance of this improvement should be interpreted cautiously. Calibration and external validation were not performed, and the results do not establish the clinical utility of C-PLAN beyond its association with survival outcomes.

The potential value of C-PLAN is that it combines several routinely available clinical and laboratory variables into a single score. This may be particularly relevant in real-world settings where more complex biomarkers are not always available. Nevertheless, C-PLAN should currently be considered a prognostic index rather than a treatment-selection tool. The present study demonstrates an association with survival outcomes but does not establish that treatment decisions based on C-PLAN improve patient outcomes. Prospective studies and external validation in independent melanoma cohorts are required before C-PLAN can be considered for routine clinical decision-making.

Our study has several strengths. First, patients were identified from two centers, providing a real-world cohort with heterogeneous clinical characteristics. Second, both PFS and OS were evaluated, and the prognostic association of C-PLAN was assessed using Kaplan–Meier analyses and multivariable Cox regression models. Third, the analyses included clinically relevant pathological and clinical variables, and the association of C-PLAN with both endpoints remained statistically significant in the primary and additional multivariable models.

Several limitations should also be considered. The retrospective design introduces the possibility of selection and information bias, and residual confounding from variables not included in the analyses cannot be excluded. The sample size was relatively modest, which may have limited the statistical power to detect associations for some variables, particularly in the OS analyses. In addition, patients were treated over a prolonged period during which the systemic treatment landscape of metastatic melanoma changed substantially. Contemporary melanoma management increasingly relies on immune checkpoint inhibitor-based therapy and, in BRAF V600-mutant disease, BRAF/MEK-targeted therapy, whereas treatment options and sequencing differed during the earlier years of our study period [3]. Consequently, treatment heterogeneity and changes in therapeutic strategies over time may have influenced survival outcomes. Although additional analyses accounted for treatment year, study center, and first-line treatment type, these adjustments cannot fully eliminate the potential effects of changes in clinical practice. Furthermore, Schoenfeld residual analyses showed no statistically significant global departure from the proportional hazards assumption in either the PFS model (global *p* = 0.060) or the OS model (global *p* = 0.057). However, mitotic activity and BRAF status showed potential departures from this assumption in the OS model, warranting caution when interpreting their corresponding hazard ratios. Finally, although our study included two centers, C-PLAN was not externally validated in an independent melanoma cohort.

## 5. Conclusions

In this two-center retrospective study, the C-PLAN index was independently associated with both progression-free survival and overall survival in patients with metastatic malignant melanoma. Patients with a C-PLAN score of ≥2 had significantly shorter PFS and OS than those with a score of 0–1. The number of metastatic sites was also independently associated with both survival outcomes in the primary multivariable analyses. The association between C-PLAN and survival persisted in additional multivariable models incorporating clinical and treatment-related variables. As a simple index based on routinely available clinical and laboratory parameters, C-PLAN may provide useful prognostic information in metastatic melanoma. However, external validation in larger, contemporary, and independent melanoma cohorts is required before its broader clinical applicability can be established.

## Figures and Tables

**Table 1:** Baseline demographic, clinical, and pathological characteristics according to the C-PLAN group.

Characteristic	C-PLAN 0–1 (n = 48)	C-PLAN ≥ 2 (n = 82)	*p* Value
Age, years			0.033
<60	28 (58.3%)	32 (39.0%)	
≥60	20 (41.7%)	50 (61.0%)	
Sex			0.020
Male	21 (43.8%)	53 (64.6%)	
Female	27 (56.3%)	29 (35.4%)	
BMI group			0.175
<30	29 (60.4%)	59 (72.0%)	
≥30	19 (39.6%)	23 (28.0%)	
Stage			0.244
I	1 (2.1%)	3 (3.7%)	
II	20 (41.7%)	26 (31.7%)	
III	12 (25.0%)	34 (41.5%)	
IV	15 (31.3%)	19 (23.2%)	
Primary tumor site			0.993
Head and neck	16 (33.3%)	27 (32.9%)	
Uvea	3 (6.3%)	4 (4.9%)	
Trunk	8 (16.7%)	14 (17.1%)	
Upper extremity	5 (10.4%)	10 (12.2%)	
Lower extremity	14 (29.2%)	22 (26.8%)	
Rectum	2 (4.2%)	5 (6.1%)	
First-line treatment			0.800
Temozolomide	22 (45.8%)	42 (51.2%)	
Dabrafenib + trametinib	12 (25.0%)	20 (24.4%)	
Ipilimumab + nivolumab	14 (29.2%)	20 (24.4%)	
BRAF mutation status			0.962
Positive	16 (33.3%)	27 (32.9%)	
Negative	32 (66.7%)	55 (67.1%)	
Mitotic rate			<0.001
<5	35 (72.9%)	30 (36.6%)	
≥5	13 (27.1%)	52 (63.4%)	
Lymphatic invasion			0.070
Absent	26 (54.2%)	31 (37.8%)	
Present	22 (45.8%)	51 (62.2%)	
Lung metastasis			0.023
Absent	28 (58.3%)	31 (37.8%)	
Present	20 (41.7%)	51 (62.2%)	
Liver metastasis			0.705
Absent	36 (75.0%)	59 (72.0%)	
Present	12 (25.0%)	23 (28.0%)	
Brain metastasis			0.005
Absent	42 (87.5%)	53 (64.6%)	
Present	6 (12.5%)	29 (35.4%)	
Bone metastasis			0.570
Absent	40 (83.3%)	65 (79.3%)	
Present	8 (16.7%)	17 (20.7%)	
Number of metastatic sites			0.022
≤2	41 (85.4%)	55 (67.1%)	
>2	7 (14.6%)	27 (32.9%)	
C-PLAN components			
Elevated CRP	14 (29.2%)	67 (81.7%)	< 0.001
ECOG PS ≥ 2	2 (4.2%)	33 (40.2%)	< 0.001
Elevated LDH	10 (20.8%)	63 (76.8%)	< 0.001
Serum albumin < 3.5 g/dL	1 (2.3%)	42 (97.7%)	< 0.001
dNLR ≥ 3	10 (20.8%)	60 (73.2%)	< 0.001

Data are presented as n (%). Categorical variables were compared using Pearson’s chi-square test or Fisher’s exact test, as appropriate. BMI, body mass index; CRP, C-reactive protein; ECOG PS, Eastern Cooperative Oncology Group performance status; LDH, lactate dehydrogenase; dNLR, derived neutrophil-to-lymphocyte ratio; C-PLAN, a composite index incorporating ECOG PS, CRP, LDH, serum albumin, and dNLR.

**Table 2:** Multivariable Cox regression analyses for progression-free survival and overall survival.

Variable	PFS HR (95% CI)	*p*-Value	OS HR (95% CI)	*p*-Value
C-PLAN group	3.193 (1.774–5.746)	<0.001	3.227 (1.910–5.455)	<0.001
Number of metastatic sites	3.482 (1.989–6.093)	<0.001	2.453 (1.538–3.912)	<0.001
BMI group	2.159 (1.243–3.753)	0.006	0.995 (0.626–1.581)	0.983
Mitotic activity	2.238 (1.305–3.839)	0.003	1.257 (0.785–2.011)	0.341
Lymphatic invasion	3.165 (1.856–5.398)	<0.001	1.235 (0.813–1.874)	0.322
BRAF status	2.060 (1.225–3.465)	0.006	1.058 (0.660–1.694)	0.816

Abbreviations: BMI, body mass index; CI, confidence interval; HR, hazard ratio; OS, overall survival; PFS, progression-free survival. Reference categories: C-PLAN score 0–1; number of metastatic sites ≤ 2; BMI ≥ 30 kg/m^2^; mitotic activity < 5; no lymphatic invasion; BRAF-positive status. Model statistics: PFS model: χ^2^ = 76.152, df = 6, *p* < 0.001. OS model: χ^2^ = 48.924, df = 6, *p* < 0.001.

## Data Availability

Data may be shared upon reasonable request.
